# Unravelling oxygen driven *α* to *β* phase transformation in tungsten

**DOI:** 10.1038/s41598-020-71650-2

**Published:** 2020-09-07

**Authors:** Ananya Chattaraj, Mohammad Balal, Ashok Kumar Yadav, Sudipta Roy Barman, Anil Kumar Sinha, Shambhu Nath Jha, Sebastien Joulie, Virginie Serin, Alain Claverie, Vijay Kumar, Aloke Kanjilal

**Affiliations:** 1 Department of Physics, School of Natural Sciences, Shiv Nadar University, NH-91, Tehsil Dadri, Gautam Buddha Nagar, Uttar Pradesh 201 314 India; 2 UGC-DAE Consortium for Scientific Research, Khandwa Road, Indore, Madhya Pradesh 452 001 India; 3 Atomic and Molecular Physics Division, Bhabha Atomic Research Centre, Mumbai, Maharashtra 400 085 India; 4 Synchrotron Utilisation Section, Raja Ramanna Centre for Advanced Technology, Indore, Madhya Pradesh 452 013 India; 5 Beamline Development and Application Section, Bhabha Atomic Research Centre, Mumbai, Maharashtra 400 085 India; 6 CEMES-CNRS and Université de Toulouse, 29 rue J. Marvig, 31055 Toulouse, France; 7 Center for Informatics, School of Natural Sciences, Shiv Nadar University, NH91, Tehsil Dadri, Gautam Buddha Nagar, Uttar Pradesh 201 314 India; 8 Dr. Vijay Kumar Foundation, 1969 Sector 4, Gurgaon, Haryana 122 001 India

**Keywords:** Condensed-matter physics, Condensed-matter physics, Materials for devices, Nanoscale materials, Theory and computation

## Abstract

Thin films of *β*-W are the most interesting for manipulating magnetic moments using spin–orbit torques, and a clear understanding of *α* to *β* phase transition in W by doping impurity, especially oxygen, is needed. Here we present a combined experimental and theoretical study using grazing incidence X-ray diffraction, photoelectron spectroscopy, electron microscopy, and ab initio calculations to explore atomic structure, bonding, and oxygen content for understanding the formation of *β*-W. It is found that the W films on SiO_2_/Si have 13–22 at.% oxygen in A15 *β* structure. Ab initio calculations show higher solution energy of oxygen in *β*-W, and a tendency to transform locally from *α* to *β* phase with increasing oxygen concentration. X-ray absorption spectroscopy also revealed local geometry of oxygen in *β*-W, in agreement with the simulated one. These results offer an opportunity for a fundamental understanding of the structural transition in *α*-W and further development of *β*-W phase for device applications.

## Introduction

There has been long-standing interest in understanding phase transformation from *α* body centered cubic (BCC) to *β* (A15) phase in W thin films^1–10^^.^ The latter has been attracting great interest due to the finding of giant spin Hall effect using spin–orbit torques (SOT)^11–16^. The phase transition however depends on film thickness, substrate, impurities, and temperature^7,17–19^. While *α* and *β* phases have been studied, understanding the effects of impurities in driving the phase transition is still lacking, in spite of much experimental work^20–22^. It is found that even a few percent of oxygen concentration can lead to a phase transition from *α* to *β* and modify the chemical and physical (electrical) properties^23,24^ of the film. Here, we present a combined experimental and theoretical study to demonstrate the role of oxygen in driving *α* to *β* phase transformation.

Shen and Mai reported the formation of *β*-W phase with 6–15 at.% oxygen at room temperature (RT) without the formation of tungsten oxide, but W remains in *α* phase when only a small amount (less than 3 at.%) of oxygen is present^17^. The *β*-W phase, however, transforms to *α*-W when annealed to 900 K presumably due to the removal of oxygen^7^. Choi found the sputter deposited W films at 20 °C in *β*-W phase when the thickness was 2.5 nm, but it transformed to *α*-W by increasing the thickness to 5 nm and was stable even at 320°C^10^. Rossnagel et al*.* deposited *β*-W film of thickness less than 45 nm on SiO_2_ substrate at RT, but it transformed to *α*-W in few days^8^, suggesting metastable behavior of *β* phase. Lee et al.^18^ studied W films on different substrates and obtained a stable *α*-W phase on thermally oxidized Si substrate above a thickness of 7 nm. Although thinner *β-*W films were grown on SiO_2_/Si substrate, thicker (40 nm) *β*-W films could be grown on Si, GaAs, MgO, and Al_2_O_3_ substrates. Clearly, there is much variation in experimental results^18,25,26^. Since thick *β*-W films are desirable for SOT applications, a proper understanding of *β* phase stabilization is extremely important. Using ab initio calculations, Al Khamees et al*.* have shown that oxygen favors tetrahedral interstitial site (TIS) in *α-*W over octahedral interstitial site (OIS)^27^. Clustering of oxygen was also favorable in the neighboring TIS sites. However, the origin of *β*-W phase and the effects of oxygen in transformation of BCC structure have not been understood yet. Our experiments suggest the presence of ~ 13–22 at.% oxygen in *β*-W, while ab initio calculations on *α*- and *β*-W phases with different O concentrations shed light on the structural transition and the involvement of disorder in *β* phase with increasing oxygen concentration.

**Figure 1:**
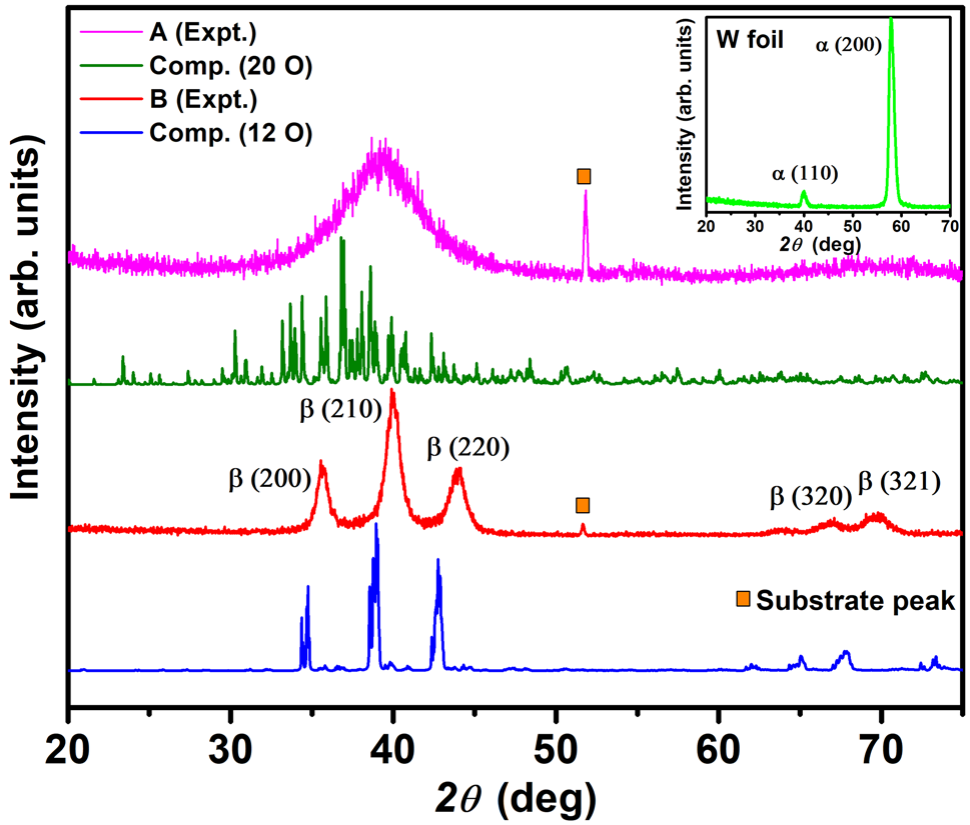
GIXRD patterns of A and B films and the calculated powder diffraction patterns from MD simulation with different concentration of oxygen in *β*-W. The inset depicts the (BCC) *α* phase in W foil. Peak marked by open box indicates a reflection from Si substrate.

We deposited ~ 35 nm and ~ 60 nm thick W films on Si(100) substrates, hereafter referred to A and B, respectively, and studied their phases by grazing incidence X-ray diffraction (GIXRD). The peaks in Fig. 1 for sample B can be assigned to the reflections from the (200), (210), and (211) planes of *β*-W (see JCPDS 03-065-6453); while A has a rather broad peak suggesting the presence of a disordered structure. Note that the observed GIXRD patterns have been reproduced even after a year, signifying the stability of the *β*-W phase. There is an additional peak at 2*θ* ≅ 52° from Si substrate, whose intensity is found to decrease with increasing film thickness; here *θ* is the Bragg angle. The XRD pattern of a W foil in *α* phase (inset of Fig. 1) signifies different reflections than those of *β*-W. These results are consistent with the calculated XRD patterns of the simulated oxygen-doped *α* and *β* phases, which will be discussed in the following.

**Figure 2:**
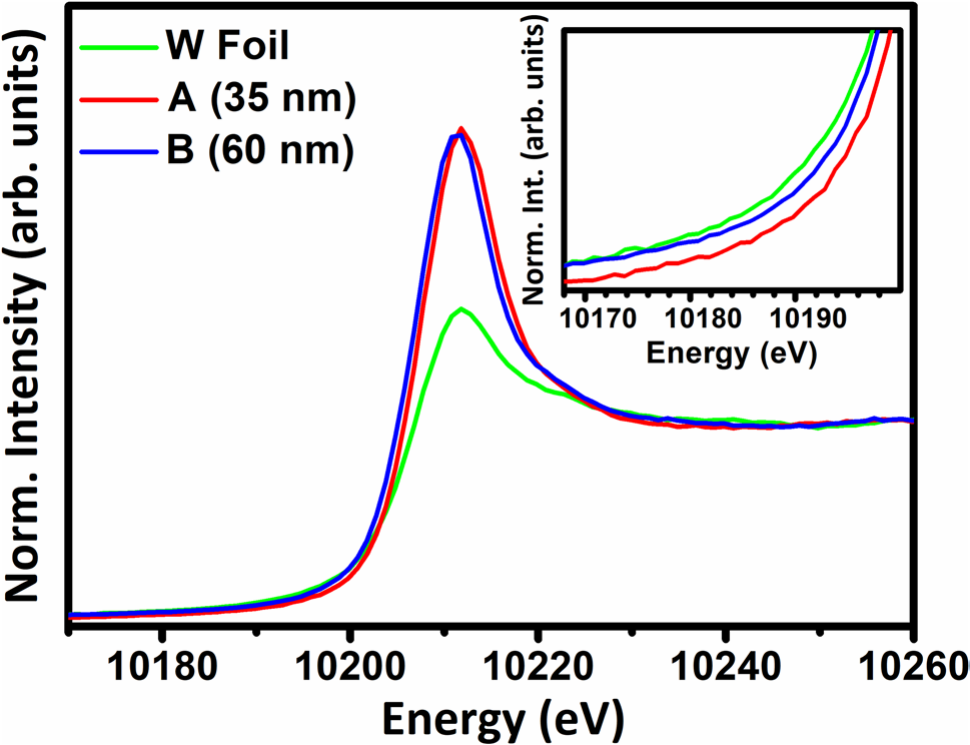
The normalized XANES spectra of A and B films at the W-*L*_3_ edge superimposed on the standard data of a W foil. The inset displays the corresponding spectral edges for comparing the oxidation behavior of W in A and B films.

The above results are supported by X-ray absorption near edge spectroscopy (XANES) at W-*L*_3_ edge, where the normalized spectra of samples A and B, shown in Fig. 2, elucidate a relative change in white lines (characteristic of the electronic transition from 2p to 5d state of W^28^). The spectra are superimposed on the standard data of a W foil (with inflection at 10,200.0 eV) for comparison. Relatively intense white lines and the blue shift in spectra of the investigated films with respect to the foil indicate a higher oxidation state of W. Since the near edge spectral shape is a signature of the electronic density of states in conjunction with local geometry and electronic property of the constituent atoms, the present observation can be attributed to the introduction of local disorder and inhomogeneous chemical environment in A and B. However, a close inspection suggests a prominent shift of the onset of W-*L*_3_ edge (inset of Fig. 2) from 10,200.0 to 10,200.8 eV in A. This corroborates to a slight increase in the oxidation behavior of W in sample A with respect to B, though the local coordination of W with oxygen atoms in any case is comparatively poor than the octahedral environment of WO_3_^29^.

**Figure 3:**
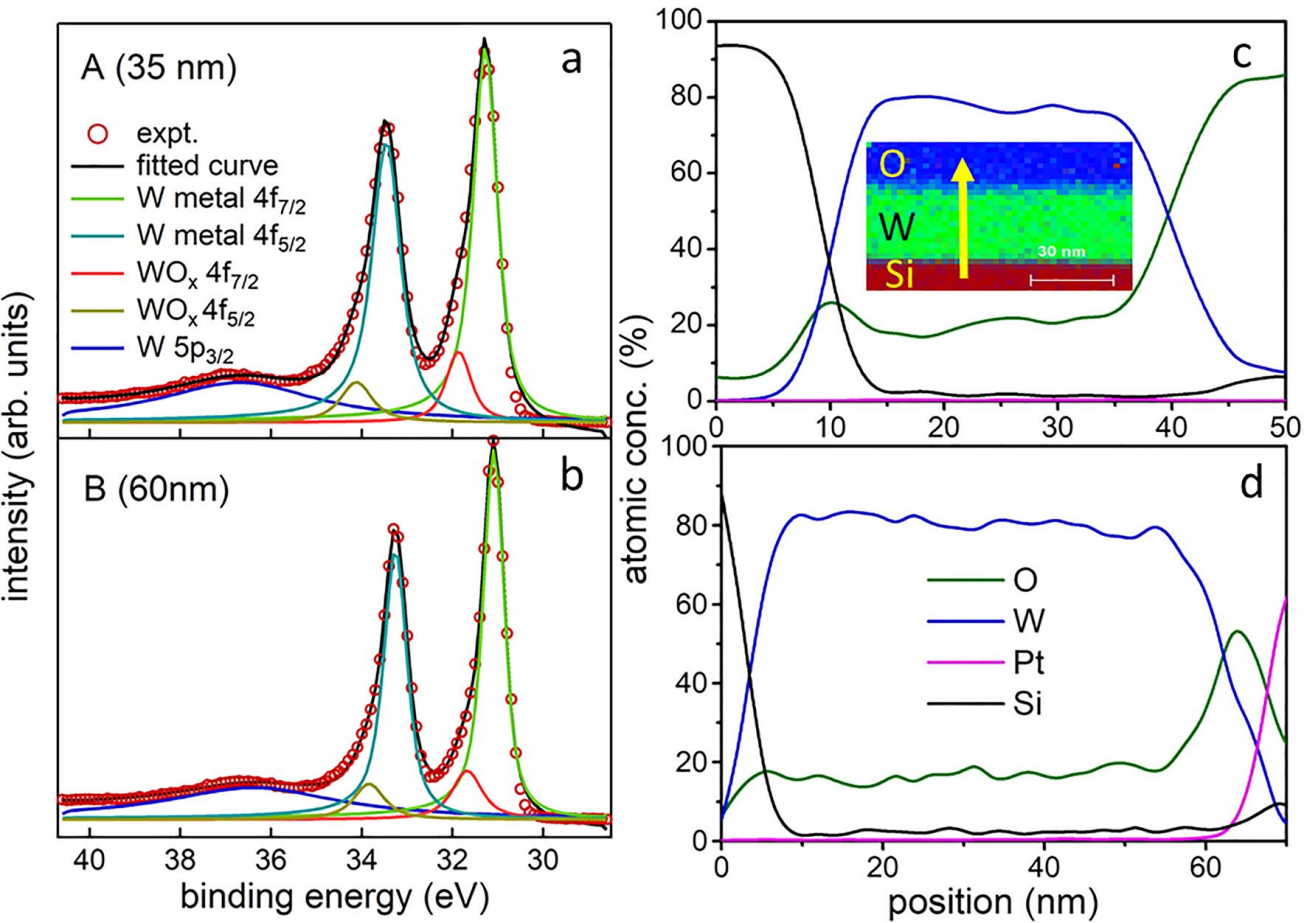
High-resolution W 4f XPS spectra for films A (**a**) and B (**b**), fitted (black line) by using a non-linear least square error minimization procedure. The fitting components are shown. Depth dependent elemental profiles of Si-*K*, W-*L*, O-*K* and Pt-*K* edges along the EDX traces across the W films of A (**c**) and B (**d**) are shown. The inset of (**c**) depicts a typical EDX map of sample A, where the scale bar is 30 nm. The upward arrow from the Si substrate to the film surface represents the direction along which EDX trace was recorded.

Further, we studied the oxidation states of W in samples A and B by using high-resolution X-ray photoelectron spectroscopy (XPS) near W 4f region, as shown in Fig. 3a,b, respectively. An asymmetry in the W 4f doublet towards high binding energy side is observed, which is indicative of oxidized W (WO_x_). In order to check whether it is not due to the well-known Doniach-Sunjic asymmetry in metals, as a first step we have performed a least square curve fitting using the same Doniach-Sunjic asymmetry parameter^30^ of 0.04 for both the spin–orbit components of W 4f, as discussed in a recent work^31^. But the fitting with single component for W 4f_7/2_ fails, showing a large asymmetry. This confirms the presence of the oxide component, and therefore an extra component representing WO_x_ was considered in the fitting. The final fitting shown in Fig. 3 is performed considering a Tougaard background, where the background parameter is also varied. The instrumental resolution is kept fixed at 0.33 eV. The fitted peak positions and the full width at half maximum (FWHM) for A and B are summarized in Table S1, in agreement with the previously reported values in the presence of oxygen^32–34^. However, the 4f_7/2_ peak position of WO_x_ was found at lower binding energy than that of WO_2_ (32.9 eV)^35^ and W^2+^ (32 eV)^36^ indicating that the valency of W is less than 2 (W^y+^, y < 2). Moreover, the relative decrease in FWHM of the 4f_7/2_ and 4f_5/2_ components in B with respect to A (Table S1) reflects the enhancement in local ordering.

We also analyzed the O1s and W4f peaks (see Fig. S1 in Supplementary Information) using their respective photoemission cross-sections, Tougaard background subtraction, and analyzer transmission function correction to determine the oxygen percentage, which is found to be 16.9% and 13.4% for A, and B, respectively. We have also performed energy dispersive X-ray spectroscopy (EDX) during TEM investigation in cross-sectional geometry. The inset of Fig. 3c displays the typical EDX map of sample A, while the arrow across the film represents the direction along which EDX trace was recorded. The depth dependent elemental profiles of Si-*K*, W-*L*, O-*K*, and Pt-*K* edges (see Fig. 3c,d) along the EDX traces across the W films of A and B indicate the variation of oxygen concentration of 16–22% and 13–20%, respectively. This is consistent with the conclusions drawn from XPS results.

The above experimental results elucidate the importance of oxygen in forming the *β*-W phase, but it is not clear why the presence of O helps the transition to occur. We have performed density functional theory calculations to understand the structural changes and bonding by optimizing atomic structures with varying oxygen concentration in *α* and *β* phases. Firstly, the optimized structures with one O at TIS and OIS in *α* and *β* supercells (for details see Fig. S2 in Supplementary Information) show TIS to be energetically more favorable in both the phases but more importantly O doping is highly favorable in *β-*W (see below) compared with the *α* phase. The lattice parameters (Table S2 in Supplementary Information) expand slightly upon O doping while the tetrahedron around O has distortions in both *α* and *β* phases. The W-W bond lengths around O in *α* change from the undoped value of 2.745 Å to 3.54 Å, 3.23 Å, 3.19 Å, and 3.0 Å, while the W–O bond lengths are of 1.97 Å. A slightly larger W–O bond length (~ 2.04 Å) is obtained in the *β* phase.

We further doped another O in both *α* and *β* supercells to find out the preferred positions and some of the optimized structures are shown in Fig. S3 in the Supplementary Information. The most favorable separation between O atoms is found to be 2.285 Å and 2.431 Å in *α* and *β* phases, respectively. It is noteworthy that in the *β* phase, the changes in the lattice parameters and bond lengths due to O doping are much less than in *α*-W with increasing number of O. In fact, the W–O bond lengths in *α*-W become similar to those in O doped *β*-W with increasing number of O leading to local transformation of an ordered *α* to a partially disordered *β* phase. This supports our experimental finding of disorder in the present *β*-W films.

We define the solution energy of O in W as $E_{S} = E_{NWO} - E_{NW} - \mu \left( O \right)$, where *E*_*NWO*_ is the total energy of the supercell containing *N* atoms of W and one O atom, *E*_*NW*_, the energy of bulk W with *N* atoms, and *µ*(*O*), the chemical potential of oxygen^27^. The calculated solution energy of O (Table 1) shows a large gain of 1.188 eV in *β-*W over the value in *α*. This important result signifies that O favors *β* structure. We further calculated the binding energy between two O atoms doped in W supercells. It is defined as $E_{b} = 2E_{1O} - E_{2O} - E_{ref}$^27^, where *E*_1O_ and *E*_2O_ are the energies of the supercells with one O only and two O atoms in TISs, respectively. *E*_ref_ is the energy of the supercell without oxygen. A positive value signifies clustering of O atoms to be favorable. The variation of *E*_*b*_ with the distance between O atoms is shown in Fig. S4 in Supplementary Information. Indeed, clustering of O atoms is favorable in both the phases (see Table 1). In *α*-W, the calculated most favorable value of *E*_*b*_ (1.417 eV) is large and it agrees with the value given by Alkhamees et al., while *E*_*b*_ is 0.339 eV in the *β*-W phase. Accordingly, clustering of O atoms is strongly favorable in *α*-W and much less in *β*-W. But interestingly, doping of two O atoms leads to 1.298 eV more gain in *β*-W compared with *α*-W, supporting again a local transformation.

**Table 1:** Calculated solution energy and binding energy of oxygen in *α*- and *β*-W. For *β*-W the TIS is inside an icosahedron while OIS represents a position slightly away from the TIS position. An interstitial site in between icosahedra was also considered, but the solution energy is − 1.147 eV.

	Solution energy of one O (eV)		Binding Energy of two O (eV)
Interstitial site	TIS	OIS	TIS
*α*-W	– 1.502	– 1.166	1.417
*β*-W	– 2.690	– 1.925	0.339

**Figure 4:**
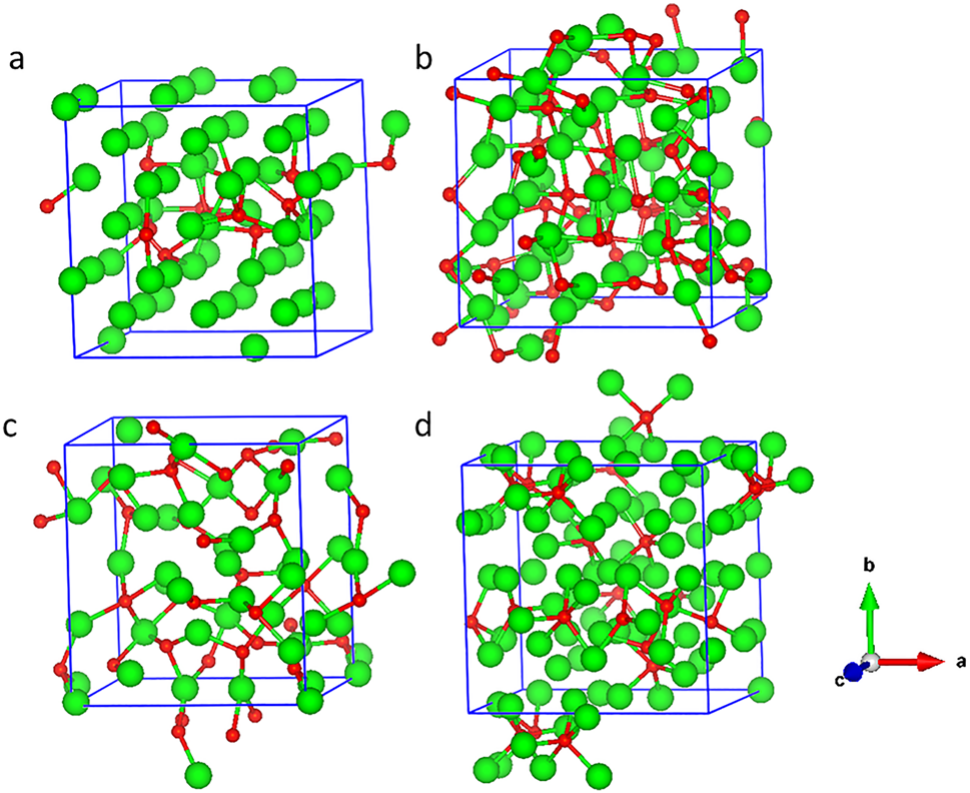
The optimized configurations obtained from ab initio MD simulations of (**a**) 3 × 3 × 3 supercell of *α*-W with 10 O, (**b**) 2 × 2 × 2 supercell of *β*-W with 12 O, (**c**) 3 × 3 × 3 supercell of *α*-W with 34 O, and (**d**) 2 × 2 × 1 supercell of *β*-W with 20 O. Green and red spheres represent W and oxygen atoms, respectively.

In *β-*W each icosahedron has 20 tetrahedra and therefore it may be possible to pack more O atoms compared with *α*-W*.* Following experimental indications, we doped 10 O in 3 × 3 × 3 *α*-W (15.6 at.%) and 12 O in 2 × 2 × 2 *β*-W (15.79 at.%) supercells randomly at TISs by keeping the separation between O atoms at least the one found optimal for two O atoms. In both the cases, the system was heated up to 973 K using ab initio molecular dynamics (MD) for 2 ps and then evolved for another 2 ps without any temperature control. In this process, we picked up a few configurations with low-lying energy and optimized them. Furthermore, we optimized the configuration after the MD run with full structure relaxation including volume and shape of the supercell. The lowest energy (optimized) structures thus obtained for *α* and *β* supercells are shown in Fig. 4a,b, respectively. The converged lattice parameters show small deviation from the cubic cells and are elongated with values of 9.65 Å, 10.23 Å, and 9.58 Å for *α*-W, and 10.32 Å, 10.43 Å, and 10.34 Å for *β*-W supercell. The elongation in the **b** direction of *α*-W supercell is large, but for *β*-W supercell, the deviation from the undoped *β*-W is only about 2–3%. We further calculated powder diffraction pattern using VESTA program for the doped *β*-W and it is consistent with the GIXRD result (see Fig. 1) of sample B except for a small shift in 2*θ*. The observed shift is most likely due to the presence of lower oxygen concentration in experiment, in addition to a slight overestimation of the lattice parameters in calculations. The difference in energy gain by O doping in the two phases decreases due to changes in the structure, but it is still 0.181 eV per O atom more in *β*-W (4.399 eV) over the value for *α*-W (4.218 eV), even for this O concentration. These results suggest a local transformation of *α*-W to *β*-W to be favorable due to the formation of W tetrahedra as well as more gain in energy in *β*-W with O doping. We also found that a large fraction of W atoms do not have any O as neighbor with ~ 16 at.% O doping. This supports our XPS results (Fig. 3a,b), as discussed above, showing the presence of neutral W and some W atoms in oxidation state of < 2.

As oxygen doping continues to be energetically favorable, we further performed ab initio MD calculations by adding 34 O atoms (38.64 at.%) in 3 × 3 × 3 *α*-W and 20 O atoms (38.46 at.%) in 2 × 2 × 1 *β*-W supercell in order to look for any ordered structure with larger oxygen concentration. Note that up to 44% oxygen has been doped in W and large spin Hall angle has been measured^12^. We followed the same procedure as above and the optimized structures are shown in Fig. 4c,d. With these O concentrations, both *α*-W and *β*-W supercells show significant expansion of the lattice parameters to 10.45 Å, 10.13 Å, and 10.55 Å for 3 × 3 × 3 *α*, and 10.45 Å, 10.82 Å, and 5.92 Å for 2 × 2 × 1 *β* supercell. The calculated powder diffraction pattern of the obtained *β*-W structure shows a large number of peaks within a 2*θ* range of 30–50°, where the peak height distribution matches well with the experimentally found broad peak in sample A with higher O concentration. It is therefore clear that an increase in O concentration leads to more disorder. The *β* phase (4.887 eV) continues to show 0.04 eV per O atom higher gain in energy with respect to the *α* phase (4.847 eV) with nearly similar content of O. More interestingly, it can be seen from the simulated powder diffraction pattern (Fig. S5 in Supplementary Information) that three peaks in the vicinity of the *β* phase reflections near 2*θ* of ~ 40° (indicated by vertical lines) along with other peaks at about 66° and 69° have evolved in the optimized *α* supercell with increasing oxygen concentration.

**Figure 5:**
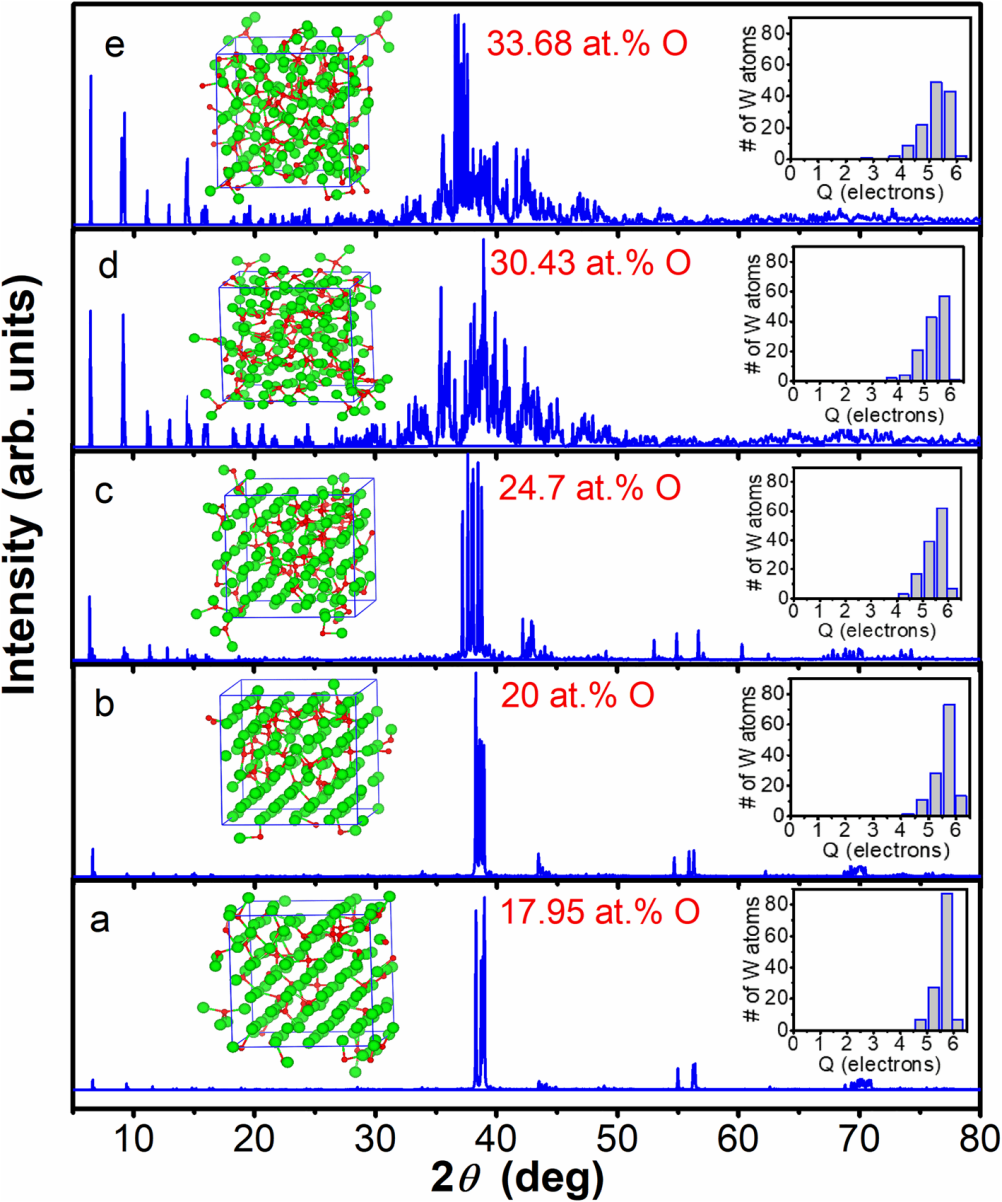
Calculated powder XRD patterns for different concentrations of oxygen in 4 × 4 × 4 supercell of *α*-W. Insets on the left side of the main peak show the corresponding atomic structure while the insets on the right show the histograms obtained from the Bader charge analysis. W (O) atoms are shown by green (red) balls. In general clustering of O is favored and for O concentration of < 25 at.% one can see regions where there is no oxygen. The number of W atoms with charge (Q) in the range of 5–5.5 e increases as the O concentration increases. There is a large fraction of W atoms that continue to have charge in the range of 5.5–6 e.

In order to further clarify the structural transition, we performed ab initio MD calculations with a larger (4 × 4 × 4) supercell of *α*-W with 128 W atoms and doped it with 17.95 at.%, 20 at.%, 24.7 at.%, 30.43 at.%, and 33.68 at.% O. The calculated optimized structures and corresponding powder diffraction patterns are shown in Fig. 5, while the lattice parameters are given in Table S3 in Supplementary Information. It can be seen that for O up to about 25 at.% (Fig. 5a–c), there are regions in the supercell where the structure has features of *α*-W and there is no oxygen. Our simulations show that oxygen favors clustering as expected from our calculations on two O atoms in the supercell, and that the region with O clustering has disorder. The corresponding powder diffraction patterns have not yet developed the features of *β* phase well. We also considered an ordered W_3_O phase following the Cr_3_O phase that is known to exist in A15 structure similar to Nb_3_Sn. However, energetically this W_3_O phase lies about 1.11 eV/atom higher in energy than the result we have obtained for the doping of about 25 at.% O in *α*-W. This result supports the fact that experimentally W_3_O A15 phase is not formed. Increasing the O concentration further to 30.43 at.%, we find that O atoms are well distributed in the supercell and all the W atoms are displaced so that the *α*-W features are no more visible (see Fig. 5d). The calculated powder diffraction pattern also develops three major peaks around the values obtained for the *β* phase suggesting that transformation of *α*-W has occurred with disorder. These results suggest that the optimal concentration of O for the structural transition of *α*-W to *β* is around 25–30 at.%, though experiments have reported the existence of *β* phase even with much less O concentration. We believe that such samples have regions of *α*-W with non-uniform distribution of low concentration of O or none, whereas *β* regions might have a higher concentration of O. This in fact agrees well with our EDX results where a significant variation in O concentration across the W film is obtained. Using 4 × 4 × 4 supercell, further calculations with higher O concentration (33.68 at.%) show that the structure develops more disorder with a broad distribution of peaks in powder diffraction, in good agreement with our experiment for sample A and similar to the results of our calculations on smaller supercell. The lattice parameters expand further (see Table S3), and the energy gain generally increases with O doping. For the 4 × 4 × 4 supercell, our calculations give the values of 4.253 eV, 4.373 eV, 4.621 eV, 4.587 eV, and 4.898 eV per O atom for 17.95, 20, 24.71, 30.43, and 33.68 at.% O concentrations, respectively. The slight decrease at 30.43 at.% O could be due to an increase in disorder in the structure and it could explain the formation of *β*-phase without further oxidation. These results help to understand different observations in experiments as the structure depends significantly on oxygen concentration. Controlling the oxygen concentration in the critical range of 25–30 at.% could lead to the formation of predominantly *β*-phase. Interestingly, for all the different O concentrations we have studied in the *α*-W phase, most of the O atoms are fourfold coordinated with a small variation in the bond length of around 2.1 Å. This again supports local structural transition in *α*-W phase with O doping. A few O atoms are found to be fivefold coordinated, while in the case of 30.43 at.% and 33.68 at.% a few of them are also threefold coordinated with slightly shorter bond lengths. A tendency to form sixfold coordinated O is seen for the highest concentration of 33.68 at.%. Figure S6 in the Supplementary Information shows the W–O, O–O, and W-W pair correlation functions *g*(r) for the case of 30.43 at.% O in the *α*-W phase. It is clear that the W–O main peak is the narrowest suggesting small variation in W–O bond lengths. The main peak in the O–O pair correlation function lies in the range of 2.4 – 2.8 Å while the W-W distribution is centered around 2.8 Å. The broad peaks suggest disorder in the structure.

We also calculated Bader charge to further understand the bonding character. Insets in Fig. 5 show the charge on different W atoms for the case of 4 × 4 × 4 supercell of *α*-W, while the charge for the case of *β*-W is shown in Fig. S7 of the Supplementary Information. It is found that the charge on O atoms has small variation and it is around 7.5 e in *α*-W and slightly higher in the case of *β*-W. It is found that in all cases, there is a large fraction of W atoms on which the charge Q lies in the range of 5.5–6 e indicating small charge transfer to O. This supports the XPS results where a strong peak is found around the neutral W value. Then there is a strong peak around 5–5.5 e which grows with increasing O concentration, and the number of W atoms having charge in the range of 5.5–6 e decreases. A small fraction of W atoms also has charge in the range of 4–4.5 e. This result is again in agreement with our XPS results which suggested the positive charge on W atoms to be less than 2. A similar behavior is obtained for the *β*-W case.

**Figure 6:**
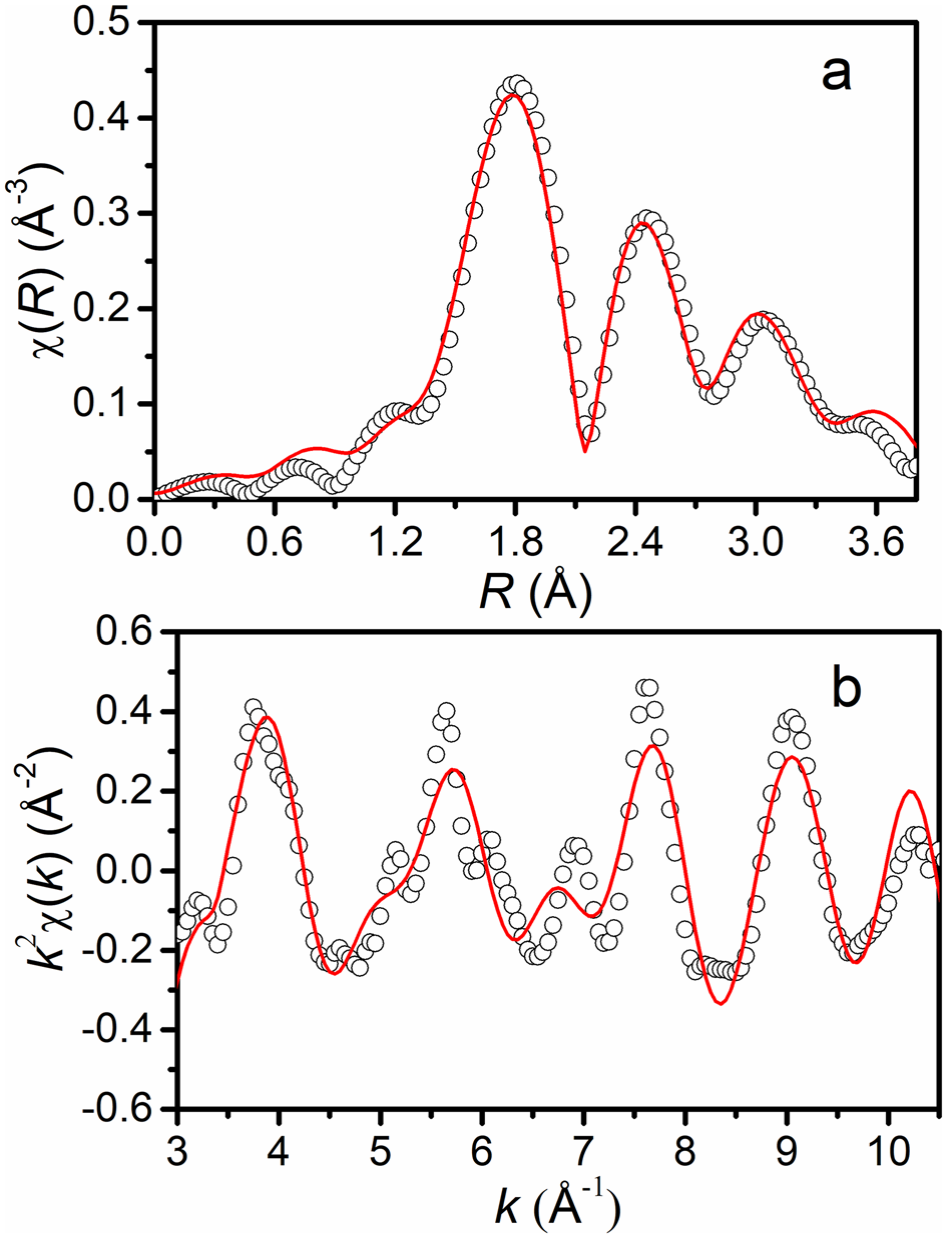
(**a**) Comparison of experimental and calculated EXAFS spectra of sample B at the W-*L*_3_ edge. The spectral features of the simulated χ(*R*) provides the average bond lengths. This is the Fourier transformation of the EXAFS spectrum in *k*^2^*χ*(*k*) space, as shown in (**b**). The solid line represents the simulated spectrum using average bond lengths obtained from calculations while the experimental EXAFS data has been shown by open circles.

In order to further confirm the structure of the observed *β* phase in the presence of oxygen, extended X-ray absorption fine structure (EXAFS) measurements have been carried out at RT. Figure 6a shows the experimental and calculated EXAFS spectra at W-*L*_3_ edge for the sample B. As discerned, the spectral features of the simulated *χ*(*R*) are fairly matching with the experimental ones, where *R* is the average distance of neighboring atom. This is actually the Fourier transformation of the EXAFS spectrum in *k*^2^*χ*(*k*) space, as shown in Fig. 6b. The average bond lengths of W–O and W-W have been estimated to be 2.11 Å and 3.74 Å, and 2.62 Å and 2.91 Å for the first and second neighbors, respectively, from the EXAFS fitting, whereas ab initio results give 2.08 Å and 3.57 Å for W–O, and 2.70 Å and 3.05 Å for W-W. These observations signify the consistency of local geometry of the *β* phase by ab initio calculation with the one determined from the EXAFS analysis. Some difference in the average bond lengths is likely to be due to difference in O concentration.

In summary, our combined experimental and theoretical study has revealed the effects of oxygen in the transformation of *α*-W to *β* phase. It is found that oxygen doping is favorable at the tetrahedral sites and it induces disorder in W such that the local environment of oxygen in *α* phase tends to become similar to that in the *β* phase. Our EDX experiments suggest variation in oxygen concentration in the range of 13–22 at.% in the samples, while the DFT calculations with ab initio molecular dynamics show clustering of oxygen to be favorable leading to local variations in oxygen concentration. Also, significantly, we find from calculations that an oxygen concentration in the range of 25–30 at.% could lead to predominantly *β* phase formation. These results and Bader charge analysis also support our XPS measurements that suggest the presence of two types of W atoms, one as in pure W and the other partially oxidized. The extracted bond lengths from EXAFS on our samples having *β*-W phase as confirmed from GIXRD, are found to be in good agreement with our DFT calculations. Oxygen doping is energetically more favorable in *β* phase and this is responsible for the transformation of *α*-W to *β*-W. Interestingly oxygen doping of about 20 at.% leads to only about 2–3% elongation in the lattice parameters for *β*, but 7–11% in the *α* phase. We believe that the present study would serve as a benchmark for further development of oxygen induced *β* phase of W.

## Methods

### Experimental

We used a 500 μm thick Si (100) wafer which was diced into pieces of area 1 × 1 cm^2^. The latter were cleaned by dipping in hot trichloroethylene, acetone, isopropanol, and deionized water in sequence for 2 min in each step. The chemically cleaned substrates were dried by blowing nitrogen. Subsequently, 99.9% pure W powder (Sigma-Aldrich) was used to grow W films at RT by electron beam deposition technique with a rate of 0.01 nm/s. The base pressure of the vacuum chamber was 5.4 × 10^–7^ Torr. The film thickness was estimated by stylus surface profilometer (Bruker, Dektak XT). The evolution of *β* phase was identified by GIXRD in Bruker D8-Discover using Cu-*K*_*α*_ radiation (*λ* = 0.154 nm). XPS was performed with a R4000 VG Scienta hemispherical electron energy analyzer and Al-*K*_α_ radiation (1486.6 eV) from a monochromatic source. Both the films were sputtered cleaned *in-situ* by 1 keV Ar^+^ ions to remove the surface oxide that is inevitable for exposure to ambient conditions. The binding energy scale of the measured kinetic energy was calibrated from the Fermi edge of a gold foil. The nature of the chemical bonding, structural distortion, and local environment of W in the presence of oxygen impurities were examined by XANES at the Angle Dispersive X-Ray Diffraction Beamline (BL-12) of Indus-2 synchrotron radiation facilities of Raja Ramanna Centre for Advanced Technology (RRCAT). Moreover, X-ray absorption spectroscopy (XAS) measurements at W-*L*_3_ edge have also been carried out at the Energy-Scanning EXAFS beamline (BL-9) at the Indus-2 Synchrotron source (2.5 GeV, 200 mA) at RRCAT^37,38^. The measurements were done in fluorescence mode using a Si drift detector (Vortex detector) in 45° geometry. The set of EXAFS data analysis program available within Demeter software package has been used for EXAFS data analysis^39^. This includes background reduction and Fourier transform to derive the *χ*(R) versus *R* spectra from the absorption spectra (using ATHENA software), generation of the theoretical EXAFS spectra starting from an assumed crystallographic structure and finally fitting of experimental data with the theoretical spectra using ARTEMIS software. The Fourier transform spectrum is phase uncorrected spectrum, where the coordination peak in Fourier transform spectrum appears at slightly lower inter-atomic distances (*R*) compared to the actual bond length. Further, elemental analysis was carried out by EDX with a 2 nm spot size during microstructure study by TEM in cross-sectional geometry. We used a (C_s_ corrected) TEM system from Tecnai-FEI operated with an acceleration voltage of 300 kV.

### Calculations

We performed ab initio calculations using Vienna ab initio simulation package (VASP)^40^ with projector augmented wave (PAW) pseudopotential^41^ method and Perdew-Burke-Ernzerhof (PBE) form of the generalized gradient approximation (GGA)^42^ for the exchange–correlation functional. The kinetic energy cutoff for the plane wave expansion of the wave function was set to be 500 eV. All the structures were completely relaxed until the absolute value of each component of the Hellmann–Feynman force on each ion dropped below 0.005 eV/Å. Both the volume and shape of the cell were allowed to change, and ions were simultaneously relaxed. We started with two-unit cells: (1) *α* (BCC, space group Im-3m) with two atoms per unit cell and (2) *β* (A15 structure, space group Pm-3n) containing eight atoms per unit cell. The Brillouin zone integrations were performed using Monkhorst–Pack scheme with 15 × 15 × 15 (9 × 9 × 9) **k**-points mesh for the *α* (*β*) unit cell. The optimized lattice parameters, 3.17 Å and 5.06 Å, are in excellent agreement with the previously reported results of 3.17 Å and 5.05 Å as well as the experimental values of 3.16 and 5.04 Å for *α*-W and *β*-W, respectively^11,27,43^^.^ The cohesive energy in *β*-W phase is 0.085 eV/atom less compared with 8.48 eV/atom obtained for the *α* phase. Therefore, *α* phase is favorable as expected. We also performed calculations for bulk *α*-W and *β*-W phases by including spin–orbit interaction. It lowers the energy by ~ 0.23 eV/atom in both the cases. The cohesive energy after spin–orbit correction is in good agreement with the experimental value of 8.9 eV/atom. As the correction is similar for the two phases, we ignored this correction for the doped cases. We used 3 × 3 × 3 supercell for *α*-W with 54 W atoms and 2 × 2 × 2 supercell for *β*-W with 64 W atoms to study the doped systems. In both the cases 5 × 5 × 5 **k**-points mesh was used for Brillouin zone integrations. The optimized lattice parameters of the undoped supercells of *α* and *β* phases were 9.51 Å and 10.12 Å, respectively, in good agreement with the experimental values. Some calculations using ab initio molecular dynamics have also been done for 4 × 4 × 4 supercell of *α*-W to understand structural transition with the doping of different concentrations of oxygen. In these calculations, the system was heated at 3000 K for 2 ps and cooled to 0 K in 3 ps. Further optimization of the structure was performed using high precision and 3 × 3 × 3 **k**-points mesh with full relaxation of the supercell and without using any symmetry.

## Supplementary information

Supplementary Information.
