# Supplementary material for: Unravelling oxygen driven α to β phase transformation in tungsten
Source: Sci Rep. 2020 Sep 7;10:14718. doi: 10.1038/s41598-020-71650-2 (PMC7477580; doi:10.1038/s41598-020-71650-2)
Supplement: Supplementary file 1 — Supplementary Information. [file 41598_2020_71650_MOESM1_ESM.docx]

**Supplementary Information**

**Unravelling oxygen driven *α* to *β* phase transformation in tungsten**

**Ananya Chattaraj^1^, Mohammad Balal^2^, Ashok Kumar Yadav^3^, Sudipta Roy Barman^2^, Anil Kumar Sinha^4^, Shambhu Nath Jha^5^, Sebastien Joulie^6^, Virginie Serin^6^, Alain Claverie^6^, Vijay Kumar^7,8^, and Aloke Kanjilal^1^**✉

^1^*Department of Physics, School of Natural Sciences, Shiv Nadar University, NH-91, Tehsil Dadri, Gautam Buddha Nagar, Uttar Pradesh 201 314, India*

^2^*UGC-DAE Consortium for Scientific Research, Khandwa Road, Indore, Madhya Pradesh 452 001, India*

^3^*Atomic and Molecular Physics Division, Bhabha Atomic Research Centre, Mumbai, Maharashtra 400085, India*

^4^*Synchrotron Utilisation Section, Raja Ramanna Centre for Advanced Technology, Indore, Madhya Pradesh-452013, India*

*^5^Beamline Development and Application Section, Bhabha Atomic Research Centre, Mumbai, Maharashtra 400 085, India*

*^6^CEMES-CNRS and Université de Toulouse, 29 rue J. Marvig, 31055 Toulouse, France*

^7^*Center for Informatics, School of Natural Sciences, Shiv Nadar University, NH91, Tehsil Dadri, Gautam Buddha Nagar, Uttar Pradesh 201 314, India*

*^8^Dr. Vijay Kumar Foundation, 1969 Sector 4, Gurgaon, Haryana 122001, India*

✉email: [aloke.kanjilal@snu.edu.in](mailto:aloke.kanjilal@snu.edu.in) (AK)

**Table S1.** XPS fitting parameters showing 4f_5/2_ and 4f_7/2_ peak positions and FWHMs of metallic W and partially oxidized W (WO_x_) in samples A and B.

| **Film** | **W 4f** | | **Peak Position**  **(eV)** | **FWHM**  **(eV)** |
| --- | --- | --- | --- | --- |
| **A** | W | 4f_7/2_ | 31.26±0.02 | 0.43±0.01 |
|  |  | 4f_5/2_ | 33.44±0.02 | 0.56±0.01 |
|  | WO_x_ | 4f_7/2_ | 31.9±0.07 | 0.5±0.06 |
|  |  | 4f_5/2_ | 34.1±0.07 | 0.7±0.06 |
|  | W | 5p_3/2_ | 36.6±0.14 | 4.06±0.4 |
| **B** | W | 4f_7/2_ | 31.08±0.01 | 0.33±0.01 |
|  |  | 4f_5/2_ | 33.24±0.01 | 0.40±0.01 |
|  | WO_x_ | 4f_7/2_ | 31.7±0.05 | 0.6±0.07 |
|  |  | 4f_5/2_ | 33.8±0.05 | 0.6±0.07 |
|  | W | 5p_3/2_ | 36.3±0.12 | 4.4±0.34 |

**Table S2**. The lattice parameters in *α* and *β* phases of W without oxygen (unit and super cells) and with one and two O atoms at the tetrahedral interstitial sites (TIS) in the supercell. The W-O bond lengths are also given.

| **W** | **Lattice parameters (Å)** | | | | **W-O Bond lengths (Å)** | |
| --- | --- | --- | --- | --- | --- | --- |
|  | Unit cell | Supercell | With 1O  a, b, c | With 2O  a, b, c | With 1O | With 2O |
| *α* | 3.17 | 9.51 | 9.55,  9.54,  9.55 | 9.55,  9.59,  9.60 | 1.97, 1.97,  1.97, 1.97 | 1.97,1.97, 2.04,  2.01, 1.99,1.99,  1.99, 1.99 |
| *β* | 5.06 | 10.12 | 10.14,  10.14,  10.14 | 10.14,  10.17,  10.16 | 2.03, 2.03,  2.03, 2.12 | 2.03, 2.05, 2.05,  2.13, 2.03, 2.05,  2.05, 2.13 |

**Table S3**. The lattice parameters of the 4×4×4 supercell of *α*-W with different concentrations of oxygen.

| **Lattice parameters (Å)** | | | | |
| --- | --- | --- | --- | --- |
| 17.95 at.% O  a, b, c (Å) | 20 at.% O  a, b, c (Å) | 24.7 at.% O  a, b, c (Å) | 30.43 at.% O  a, b, c (Å) | 33.68 at.% O  a, b, c (Å) |
| 13.040, 13.072, 13.349 | 13.069, 13.155, 13.423 | 13.360, 12.964, 13.799 | 13.483, 13.601, 13.709 | 13.732, 13.706, 13.723 |


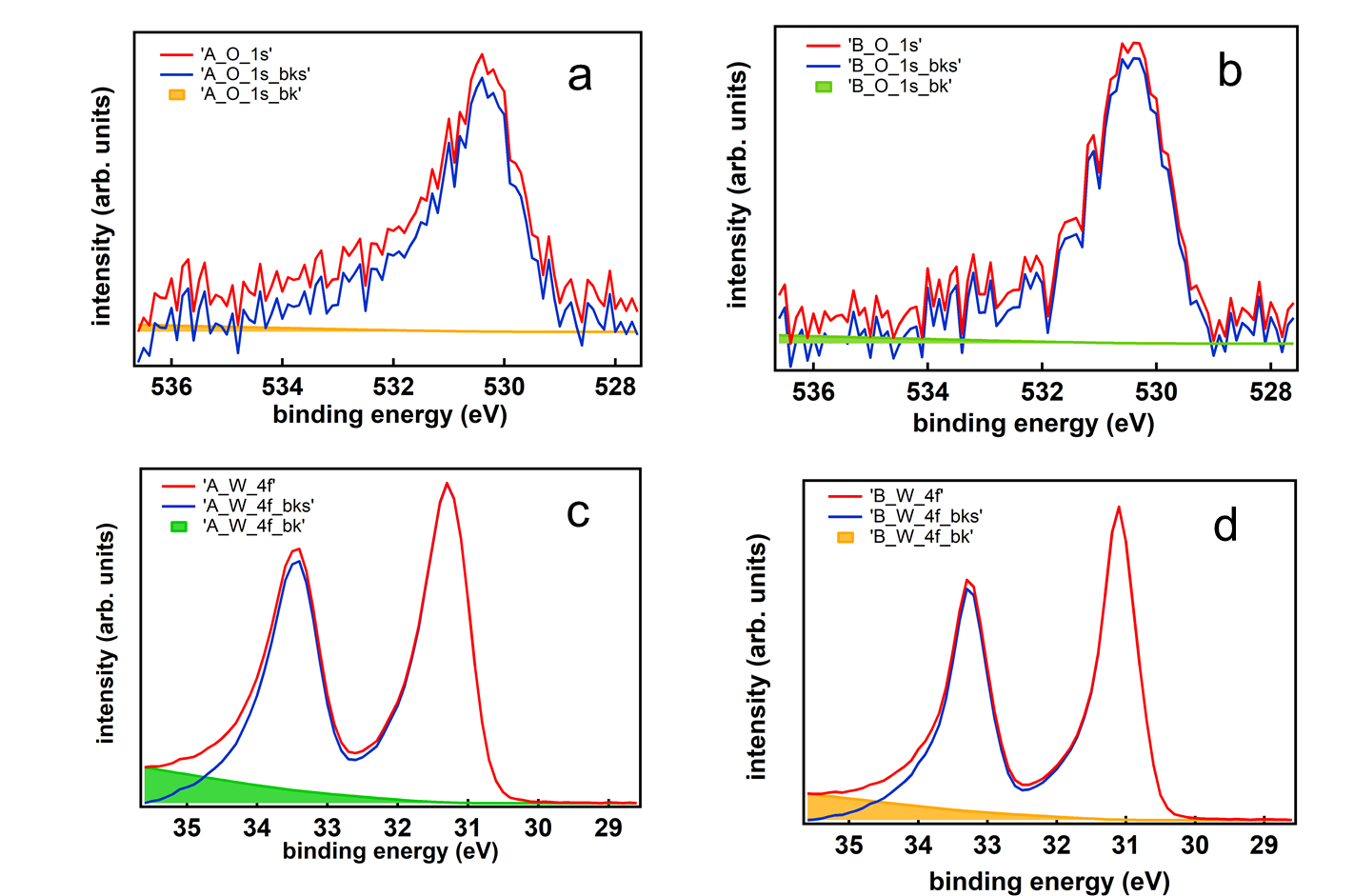


**Figure S1.** XPS spectra for O 1s of sample A (a) and B (b), and correspondingly (c) and (d) for W 4f peak using Tougaard background subtraction (shaded) to estimate the atomic percentage of oxygen in A and B films. Red line signifies the experimental raw data, where blue line represents the background subtracted spectra.


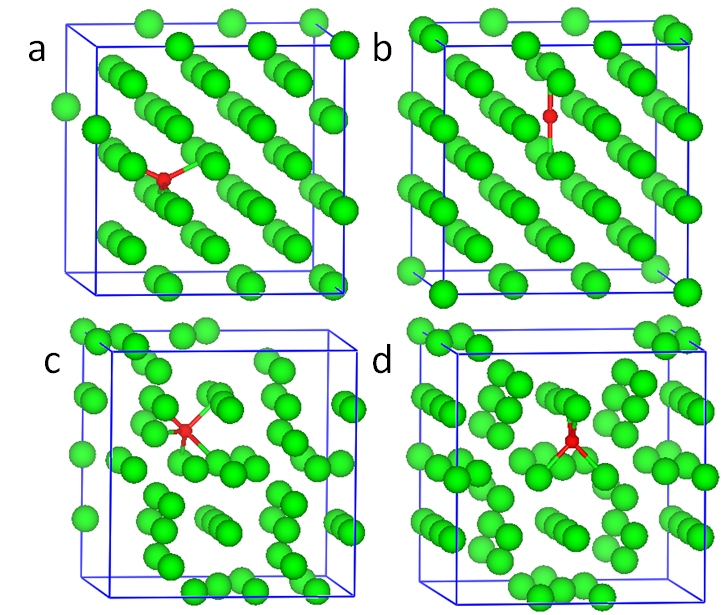


**Figure S2.** Optimized atomic structures of (a) tetrahedral interstitial site and (b) octahedral interstitial site in *α*, whereas (c) and (d) for the corresponding sites in *β*-W phase. Green (red) balls show W (O) atoms.


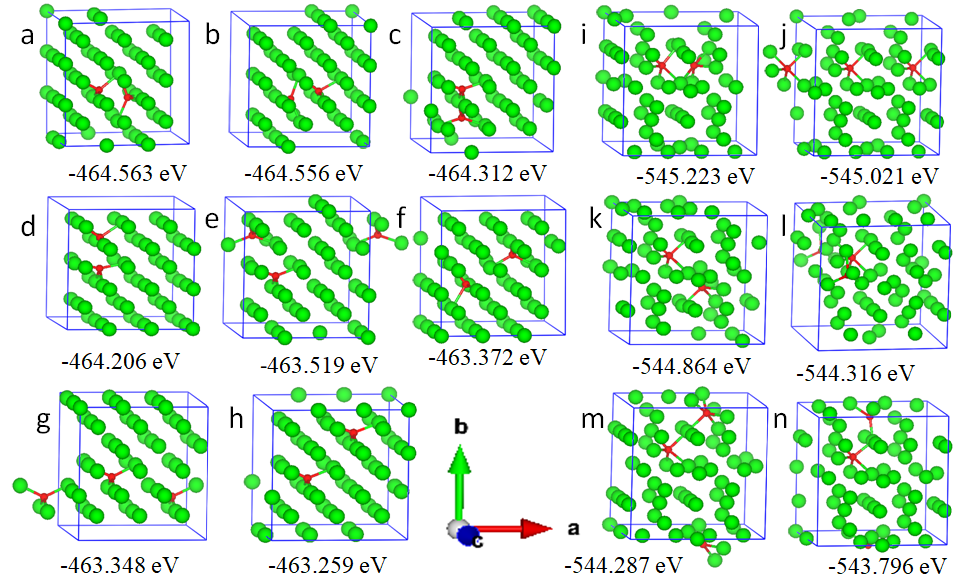


**Figure S3.** Different configurations with two O atoms in *α* (a-h) and *β* (i-n) supercells. The total cohesive energy is given below each figure. Green (red) balls show W (O) atoms.


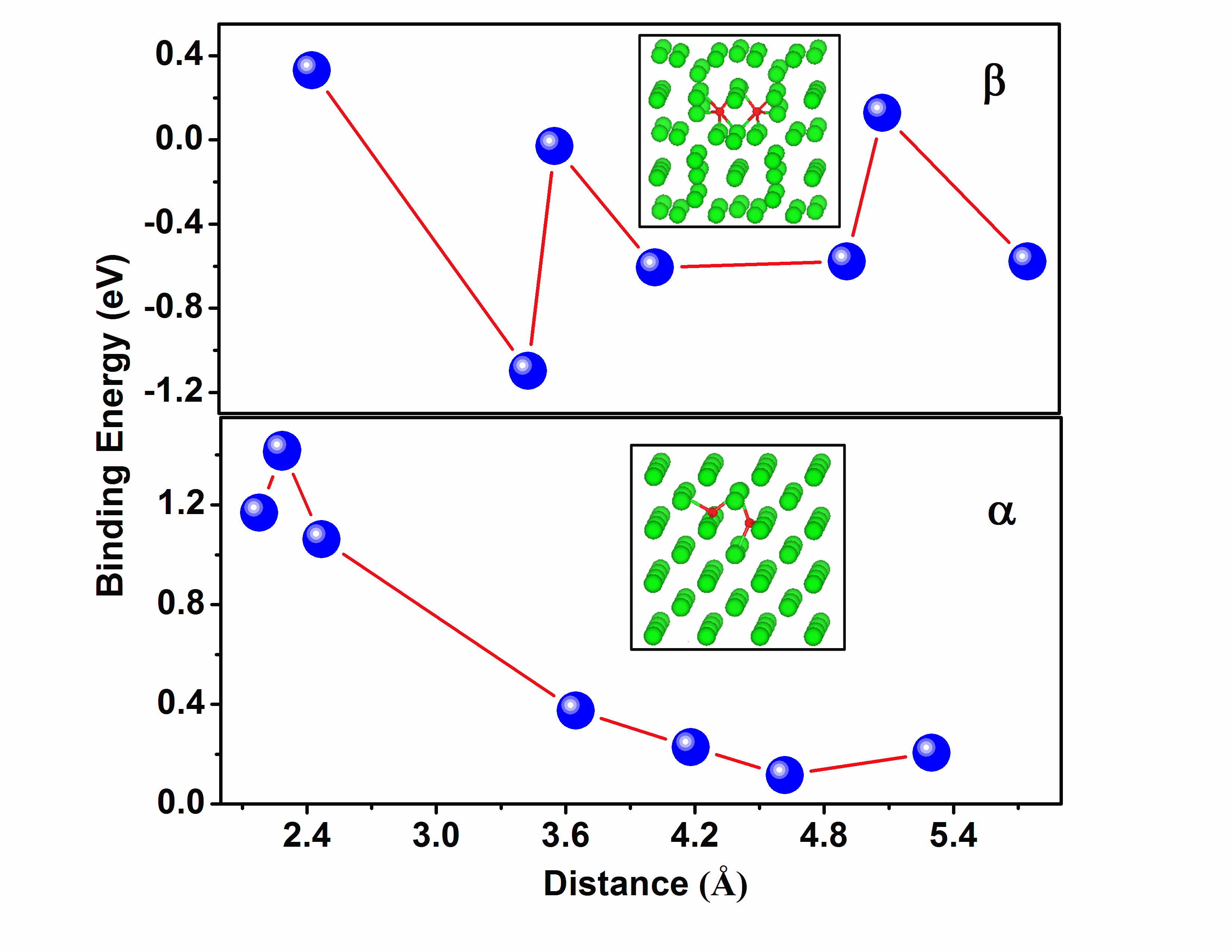


**Figure S4.** The variation of the binding energy between two O atoms in *α* and *β* phases. The insets show the atomic structures for the lowest energy configuration. Green (red) balls show W(O) atoms.

**Figure S5.** Calculated powder diffraction patterns for different concentrations of oxygen in *α* phase using 3×3×3 supercell show significant shift towards lower 2*θ* value with increasing oxygen concentration for which the lattice parameters are affected more significantly than in *β* phase. Oxygen doping also leads to splitting of the peaks of the pure *α*-W. The black vertical lines are guide to the eyes to follow the gradual evolution of peaks as oxygen concentration is changed.





**Figure S6.** The partial pair correlation functions for W-O, O-O, and W-W in the case of 30.43 at.% O in 4×4×4 supercell of *α*-W.


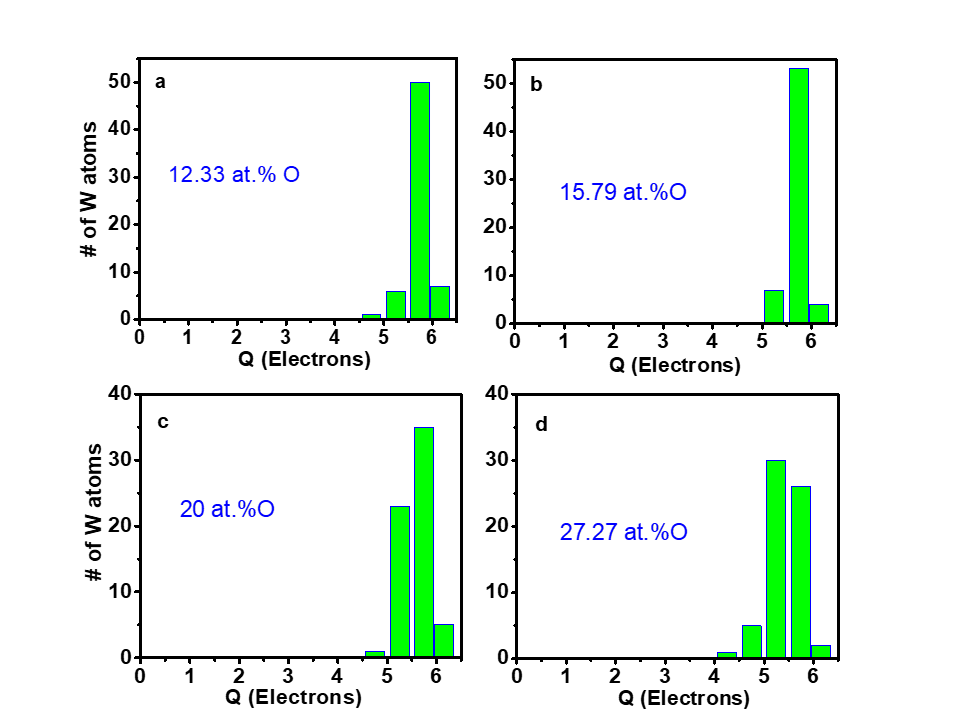


**Figure S7**. Bader charge Q on W atoms for different concentration of O in 2×2×2 supercell of *β*-W.
